# Rapid Detection of Pesticide Residues in Paddy Water Using Surface-Enhanced Raman Spectroscopy

**DOI:** 10.3390/s19030506

**Published:** 2019-01-26

**Authors:** Shizhuang Weng, Wenxiu Zhu, Ronglu Dong, Ling Zheng, Fang Wang

**Affiliations:** 1 National Engineering Research Center for Agro-Ecological Big Data Analysis & Application, Anhui University, Hefei 230601, China; zhuzwx1995@163.com (W.Z.); xyx_cm0018@163.com (F.W.); 2 Institute of Intelligent Machines, Chinese Academy of Sciences, Hefei 230031, China; dongrl@mail.ustc.edu.cn

**Keywords:** pesticides, SERS, paddy water, rapid detection

## Abstract

Pesticide residue in paddy water is one of the main factors affecting the quality and safety of rice, however, the negative effect of this residue can be effectively prevented and reduced through early detection. This study developed a rapid detection method for fonofos, phosmet, and sulfoxaflor in paddy water through chemometric methods and surface-enhanced Raman spectroscopy (SERS). Residue from paddy water samples was directly used for SERS measurement. The obtained spectra from the SERS can detect 0.5 mg/L fonofos, 0.25 mg/L phosmet, and 1 mg/L sulfoxaflor through the appearance of major characteristic peaks. Then, we used chemometric methods to develop models for the intelligent analysis of pesticides, alongside the SERS spectra. The classification models developed by K-nearest neighbor identified all of the samples, with an accuracy of 100%. For the quantitative analysis, the partial least squares regression models obtained the best predicted performance for fonofos and sulfoxaflor, and the support vector machine model provided optimal results, with a root-mean-square error of validation of 0.207 and a coefficient of determination of validation of 0.99952, for phosmet. Experiments for actual contaminated samples also showed that the above models predicted the pesticide residue values with high accuracy. Overall, using SERS with chemometric methods provided a simple and convenient approach for the detection of pesticide residues in paddy water.

## 1. Introduction

Rice is one of the most important grain crops around the world, serving as a staple food for 50% of the world’s population [1]. Rice cultivation is mostly distributed around Asia, with China ranking first in the annual rice production worldwide [2]. Pest infestation considerably decreases rice yield during planting [3]. Pests can be well controlled in rice through the reasonable and appropriate application of pesticides. In China, the family-based production mode is still widespread; thus, the management of pesticide use for pest control and prevention remains difficult [4]. Pesticide overuse can lead to the accumulation of pesticide residues, which can be absorbed by the rice. The negative effects of these residues can be prevented or reduced through early detection [5,6]. Conventional detection methods, such as chromatography and mass spectrometry, are accurate, but they require complex pretreatment, long detection times, and large reagent consumption [6,7,8]. Thus, they are unsuitable for widespread pesticide residue. 

Surface-enhanced Raman spectroscopy (SERS) has the outstanding features of fingerprint characteristics, simple pretreatment, fast spectral measurement, and weak signal interference of water [9,10], which are suitable for detecting pesticide residues in water. Many researchers have used SERS to detect aflatoxins in maize [11], phorate and fenthion pesticides in apple skin [12], and thiabendazole pesticides in rape [13]. Along with SERS, chemometric methods are often used for solving spectral denoising [14,15], feature extraction [16], and modeling [16,17]. These methods can easily extract information from spectra, thereby greatly accelerating SERS detection. The regression relationship between the concentration and the spectrum of single and mixed food preservatives is usually predicted by using partial least squares regression (PLSR) [18]. In one study, K-near neighbor (KNN) was used to establish a classification model for chlorpyrifos residue in tea-poisoning, with an accuracy of 90.84–100.00% [19]. Chen et al. developed a support vector machine (SVM) classification model with an accuracy of 96.996% for chicken androgen screening [20]. Another study adopted dynamic SERS and random forest (RF) to detect fenthion in fruit and vegetable skins; the detection lower limit was 0.05 mg/L, and the root-mean-square error was 0.0101 mg/L [21]. A silicate prediction model for drug-induced respiratory toxicity was developed by the naive Bayesian (NB) classifier, with an overall prediction accuracy of 91.8% [22]. 

In this study, three pesticides (fonofos, phosmet, and sulfoxaflor) which are commonly used in rice fields were selected as detecting objects. The residues of these pesticides in paddy water were detected by SERS. The spectra were obtained with a portable spectrometer (B&WTEK iRaman-785plus, B&W TEK, Newark, DE, USA). Chemometric methods, such as PLSR, SVM, KNN, RF, and NB were used for the qualitative and quantitative analysis of different pesticides. To the best of our knowledge, only a few similar works have been conducted.

## 2. Materials and Methods

### 2.1. Materials

Fonofos (99.9%), phosmet (99.96%), and sulfoxaflor (99.9%) were obtained from the National Institute of Merology, China. Cetyltrime-thylammonium bromide (CTAB), hydrogen tetrachloroaurate, trisodium citrate, L-ascorbic acid, sodium borohydride, and silver nitrate were purchased from the Aladdin Industrial Corporation (Shanghai, China). 

### 2.2. Sample Preparation 

Fifteen water samples were collected from Feixi rice-base in Hefei. Fonofos, phosmet, or sulfoxaflor was added to a water sample. Fonofos concentrations in the paddy water were 10 mg/L, 5 mg/L, 2 mg/L, 1 mg/L, and 0.5 mg/L; phosmet concentrations were 10 mg/L, 5 mg/L, 2 mg/L, 1 mg/L, 0.5 mg/L, and 0.25 mg/L, and sulfoxaflor concentrations were 20 mg/L, 15 mg/L, 10 mg/L, 5 mg/L, 2 mg/L, and 1 mg/L. Then, 1.5 mL of each type of contaminated water sample was centrifuged at 4000 rpm for 3 min, and the supernatant was used for the spectrum measurement.

The contaminated paddy water samples were obtained from the Center of Agricultural Products’ Quality and Safety, Anhui Academy of Agricultural Sciences. Actual values were obtained using a GC–MS instrument (Thermo Fisher, TSQ8000EVO, Waltham, MA, USA), and the detection procedure was performed according to Huang’s work [23]. GC–MS results were provided by the Center of Modern Experimental Technology, Anhui University. Fonofos, phosmet, and sulfoxaflor residues in the actual samples were 1.05–9.73 mg/L, 0.22–4.96 mg/L, and 0.95–10.03 mg/L (Table S1).

### 2.3. SERS Measurement

**Figure 1:**
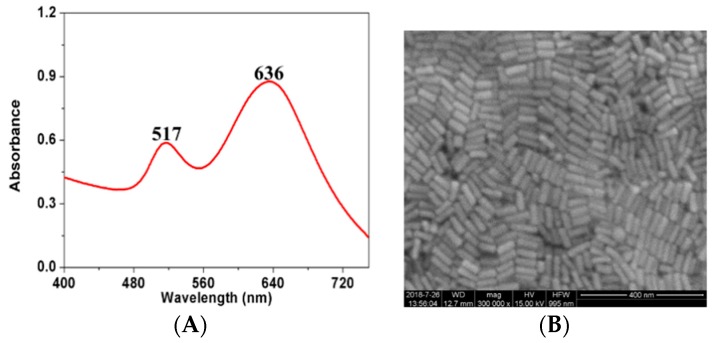
UV–Vis spectrum of the prepared gold nanorods’ (GNRs) colloid (**A**) and SEM image of GNRs (**B**).

Gold nanorods (GNRs) were synthesized through a seed-mediated growth method previously developed by Nikoobakht [24]. The synthesis and selection of GNRs of a particular aspect ratio are shown in the supporting information. The absorption spectra of the selected GNRs were recorded on an ultraviolet-visible (UV–Vis) spectrometer (UV-2600, Shimadzu, Kyoto, Japan). Scanning electron microscopy (SEM) images were obtained with a JSM 7500F microscope (JEOL Ltd., Tokyo, Japan). The morphologies of the GNRs were surveyed by using the SEM images. The GNRs exhibited two plasmon resonance bands of 517 and 636 nm (Figure 1 and Figure S3), which correspond to electron oscillations along the short and long axes of the nanorods. The GNRs were uniform and ordered.

The GNRs’ sol-solution was centrifuged at 8000 rpm for 10 min to obtain a gray colloid and 2 μL of GNRs’ colloid was dropped on a silicon chip. After drying the droplet, 2 μL of the testing water sample was dropped on the GNRs film. When the solvent was evaporated to dryness, the spectra were obtained with a portable Raman spectrometer (B&WTEK i-Raman785^®^Plus, B&WTEK, Newark, DE, USA) equipped with a 785 nm laser with a power of 150 mW. The measurement was performed with 3 scans and an exposure time of 5 s, and the spectral resolution was 2 cm^−1^ in the Raman shift range of 350 cm^−1^ to 1750 cm^−1^. Two spectra for each sample were collected as the representative spectra. 

### 2.4. Spectral Analysis

The obtained spectra were combined through the chemometrics method for the development of models for species identification and for the quantitative analysis of pesticides in paddy water. First, the spectra were baseline-corrected using polynomial fitting methods. Then, SVM, KNN, RF, and NB were adopted for the establishment of a classification model. The accuracy was used for the evaluation of the model’s performance. Regression models used for obtaining the concentration information of pesticide residues were developed through PLSR, SVM, and RF. The prediction performance of the models was quantitatively evaluated with the root-mean-square error (RMSE) and the coefficient of determination (R^2^). The spectral data were divided into two sets; 80% of the spectra for each category were selected as the calibration set for training the models, and the remaining 20% were used as the validation set for evaluating the models (Table S2). The data analysis and chemometric methods were performed in MATLAB 2013a (Mathworks Inc., Natick, MA, USA). The free SVM toolbox (developed by Prof. Zhiren Lin, National Taiwan University, Taipei, Taiwan) and KNN toolbox were used in the development of the models.

## 3. Results and Discussion

### 3.1. Spectra of Fonofos, Phosmet, and Sulfoxaflor in Paddy Water

**Figure 2:**
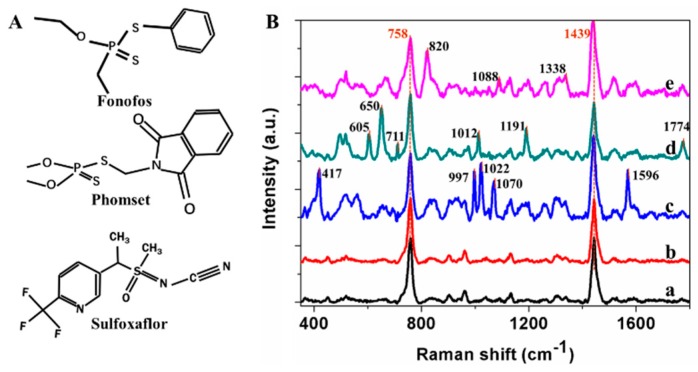
The chemical structures (**A**) of fonofos, phosmet, and sulfoxaflor, and the surface-enhanced Raman spectroscopy (SERS) spectra (**B**) of GNRs (**a**), paddy water (**b**), fonofos (**c**), phosmet (**d**), and sulfoxaflor (**e**).

The feasibility of pesticide detection in paddy water was determined by using the SERS technique, and the spectra of GNRs, paddy water, and water samples with 10 mg/L fonofos, 10 mg/L phosmet, and 20 mg/L sulfoxaflor were measured and shown in Figure 2. After a step of simple centrifugation, the aforementioned samples were directly used for the SERS measurement, which was very convenient. Firstly, the spectra of GNRs (Figure 2Ba) and water (Figure 2Bb) were consistent, and the water samples did not introduce interference. The peaks at 758 and 1439 cm^−1^ should be assigned to the CTAB residue [25]. As seen in the representative spectra of fonofos, phosmet, and sulfoxaflor in water, the spectra of different pesticides had obvious differences. 

The characteristic peaks of the SERS spectra reflect the vibration and rotation information of the analyte molecule; this information is the basic criterion for the detection of a substance. According to Vongsvivut’s work [26], the peak of fonofos (Figure 2A,Bc)) at 417 cm^−1^ is due to the scissor deformation of P–O–C, and the peaks of 997 and 1022 cm^−1^ are attributed to the in-plane deformation of the CCC and C–H in phenyl. The peaks at 1070 and 1596 cm^−1^ are ascribed to the stretching mode of S–C and C=C, respectively. For the spectra of phosmet (Figure 2A,Bd)), the deformation of C=O is at 711 cm^−1^, while the benzene ring breathing is at 1012 cm^−1^. The Raman band at 1191 cm^−1^ is due to the antisymmetric stretching mode of C–N, and the peak at 1774 cm^−1^ can be attributed to the stretching mode of C=O [27]. For sulfoxaflor (Figure 2A,Be)), the peaks at 820, 1088 and 1338 cm^−1^ are ascribed to the stretching vibrations of the pyridine skeleton, N=S=O, and the pyridine skeleton, respectively [28]. Considering these indicative peaks, SERS can be used for the detection of fonofos, phosmet, and sulfoxaflor in paddy water.

**Figure 3:**
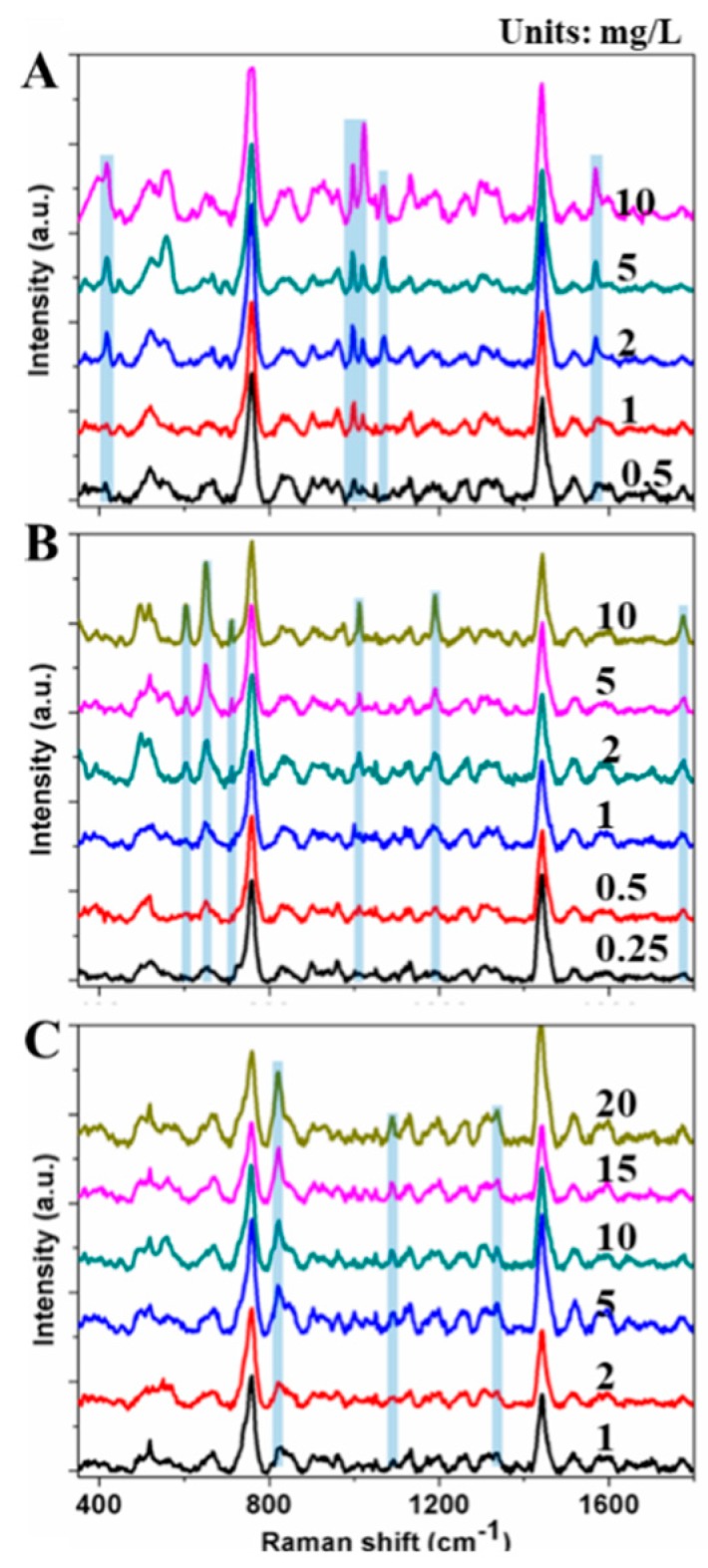
SERS spectra of fonofos (**A**), phosmet (**B**), and sulfoxaflor (**C**) of different concentrations in paddy water.

Three pesticides of different concentrations in paddy water were detected by SERS, and the representative spectra are shown in Figure 3. The intensities of the characteristic peaks of the pesticides were positively related to their concentrations in water. The result preliminarily proved that SERS can be used for the quantitative analysis of residues. For spectra of 0.5 mg/L fonofos, 0.25 mg/L phosmet, and 1 mg/L sulfoxaflor, most of the characteristic peaks disappeared; thus, SERS can detect pesticide residues of these magnitudes.

**Figure 4:**
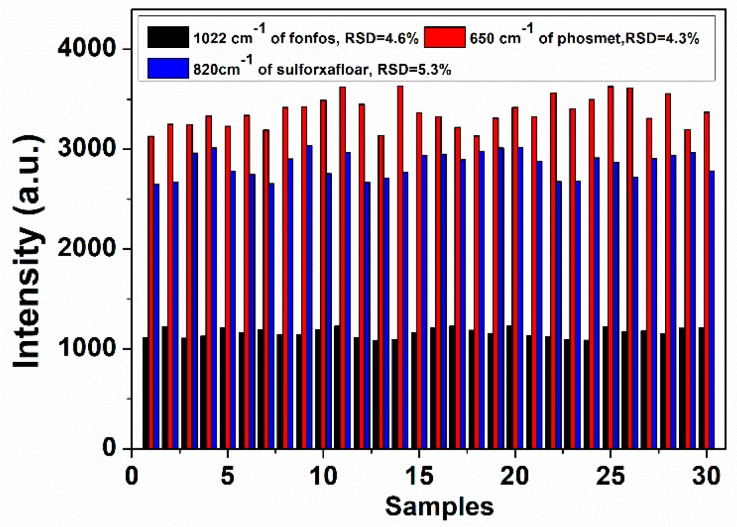
The intensity deviation of the peak at 1022 cm^−1^ for 2 mg/L fonofos (black), the peak at 650 cm^−1^ for 5 mg/L phosmet (red), and the peak at 820 cm^−1^ for 10 mg/L sulfoxaflor (blue). The selection of representative peaks was based on the intensity.

The intensity deviation of the SERS peak for different samples with pesticides of the same concentration is shown in Figure 4. The intensity had small differences, with a relative standard deviation of 4.3–5.3%. The result showed good reproducibility of SERS with GNRs and demonstrated the feasibility of accurate analysis. Meanwhile, considering that the peaks of CTAB in the SERS were stable, the band at 758 cm^−1^ was selected as an internal standard for the calibration of the band intensity (Figure S1, Supporting Information). 

### 3.2. Classification of Fonofos, Phosmet, and Sulfoxaflor in Paddy Water

For the detection of analytes, the recognition of the residue species is a key and primary step. Considering the fingerprint characteristic of the SERS technique, the recognition of species is easy for experienced experts, but it is still difficult for general personnel. Here, many multivariate analysis methods (VM, KNN, RF, and NB) were used in the development of a classification model for the recognition of pesticides in paddy water. The samples of the same pesticide residue and of the same concentration were labeled as one class, and all of the samples were divided into 17 classes. For example, the concentrations of fonofos were 10 mg/L, 5 mg/L, 2 mg/L, 1 mg/L, and 0.5 mg/L, and the solution samples with fonofos were classified into five classes. The classification results are shown in Table 1. NB obtained the worst result. The models developed by SVM and RF can identify the pesticides with an accuracy of 100% in the samples included in the calibration set. Meanwhile, the SVM and RF models obtained classification accuracies of 84.70% and 94.12%, respectively, from the samples in the validation set. This phenomenon might have occurred because the two models recognize sulfoxaflor and fonofos with low accuracy. However, KNN models can recognize all of the samples with an accuracy of 100%. KNN is a simple classification method with the time complexity of ***O(n)*** and is suitable for nonlinear classification, having the advantages of high accuracy and insensitivity to outliers [29]. These reasons may make the KNN model superior to the other models. Therefore, SERS and KNN can be adopted for the fast recognition of pesticides in paddy water [30].

### 3.3. Quantitation of Fonofos, Phosmet, and Sulfoxaflor in Paddy Water

**Figure 5:**
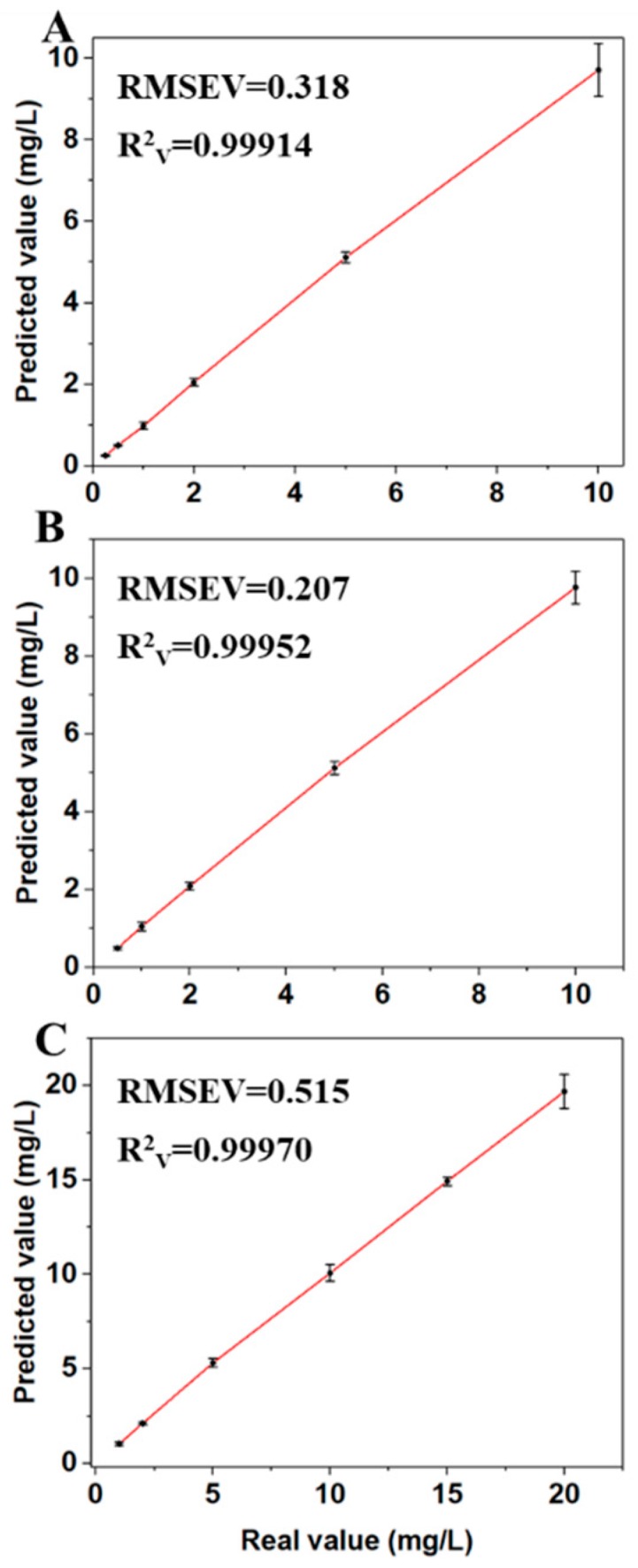
The predicted values and deviations of fonofos (**A**), phosmet (**B**), and sulfoxaflor (**C**) of different concentrations in paddy water, by using the optimal models.

The intelligent and quantitative analysis of residues in paddy water was achieved by using PLSR, SVM, and RF to build the regression models. The predicted results of the models are shown in Table 2. The models with the lowest error for fonofos and sulfoxaflor were developed by PLSR. The predicted results were RMSEC = 0.277, R^2^_C_ = 0.99981, RMSEV = 0.318, R^2^_V_ = 0.99914, RMSEC = 0.520, R^2^_C_ = 0.99992, RMSEV = 0.515, and R^2^_V_ = 0.99970, respectively. The SVM models for phosmet obtained the best result of RMSEC = 0.028, R^2^_C_ = 0.99998, RMSEV = 0.207, and R^2^_V_ = 0.99952. The detailed values of the models of best performance are shown in Figure 5. The concentrations of pesticide residues were predicted. The error was small, and the predicted values for the samples of adjacent concentrations were not overlapping. Therefore, the above optimal models are feasible for the detection of pesticide residues in paddy water with SERS.

The spectra of actual contaminated samples were measured (Figure S2 in Support information), and the predicted value was obtained using the optimal models, as shown in Table 3. The deviation was small between the predicted value and the reference value, with an relative standard deviation (RSD) of 1.22–9.38% and a recovery of 91.67–109.38%. These results demonstrated that the proposed method was feasible for the detection of pesticides in actual samples. 

## 4. Conclusions

The study explored a rapid detection method for pesticide residues in paddy water by using SERS alongside chemometric methods. Paddy water samples with fonofos, phosmet, and sulfoxaflor were directly used for SERS measurement. The obtained spectra showed that SERS can detect 0.5 mg/L fonofos, 0.25 mg/L phosmet, and 1 mg/L sulfoxaflor. The models used for the qualitative and quantitative analysis of the pesticides in the paddy water were developed through SVM, KNN, RF, NB, and PLSR. The KNN models can recognize all of the samples with an accuracy of 100%. In the quantitative analysis, the PLSR models obtained the best predicted performance for fonofos and sulfoxaflor. In the SVM model, the optimal results were RMSEV = 0.207 and R^2^_V_ = 0.99952 for phosmet. The results from the actual contaminated samples showed that the above models predicted the pesticide residue values with an RSD of 1.22–9.38% and a recovery of 91.67–109.38%. The detailed results showed that the residue concentrations calculated by the models are nearly equal to the actual concentrations. Consequently, SERS and chemometric methods can provide a novel approach for the recognition and detection of pesticide residues in paddy water.

## Figures and Tables

**Table 1:** Classified results of pesticides in paddy water using different methods.

Methods	ACC_C_ (%)	ACC_V_ (%)	Pesticides	ACC_C_ (%)	ACC_V_ (%)
SVM	100	84.70%	fonofos	100.0	80.0
			phosmet	100.0	100.0
			sulfoxaflor	100.0	73.3
KNN	100%	100%	fonofos	100.0	100.0
			phosmet	100.0	100.0
			sulfoxaflor	100.0	100.0
RF	100%	94.12%	fonofos	100.0	88.0
			phosmet	100.0	100.0
			sulfoxaflor	100.0	93.0
NB	81%	83%	fonofos	82.40	88.0
			phosmet	92.60	96.7
			sulfoxaflor	67.30	66.7
**ACC_C_: accuracy for the calibration set, ACC_V_: accuracy for the validation set.**					

**Table 2:** The quantitation of pesticides in paddy water using the models developed by PLSR, SVM, and RF.

Pesticides	Methods	RMSEC	R^2^_C_	RMSEV	R^2^_V_
fonofos	PLSR	0.277	0.99981	0.318	0.99914
	SVM	0.106	0.99993	0.347	0.99906
	RF	0.281	0.99979	1.026	0.99723
phosmet	PLSR	0.105	0.99994	0.257	0.99940
	SVM	0.028	0.99998	0.207	0.99952
	RF	0.3012	0.99983	1.1502	0.99733
sulfoxaflor	PLSR	0.520	0.99992	0.515	0.99970
	SVM	0.206	0.99997	0.969	0.99944
	RF	0.5298	0.99992	1.5229	0.99912

**Table 3:** The detection of the actual contaminated samples using the optimal models.

Pesticides	Reference Value (mg/L)	Mean Predicted Value (mg/L)	Relative Deviation (%)	Recovery (%)
fonofos	9.73	9.33	4.29	104.29
	9.54	9.12	4.61	104.61
	4.76	4.91	3.05	96.95
	1.97	1.86	5.91	105.91
	1.05	0.96	9.38	109.38
phosmet	4.96	5.07	2.17	97.83
	2.21	2.11	4.74	104.74
	0.93	1.01	7.92	92.08
	0.55	0.54	1.85	101.85
	0.22	0.24	8.33	91.67
sulfoxaflor	10.03	9.88	1.52	101.52
	9.56	9.34	2.36	102.36
	4.86	4.92	1.22	98.78
	2.03	2.11	3.79	96.21
	0.95	0.91	4.40	104.40

## Supplementary Materials

The following are available online at http://www.mdpi.com/1424-8220/19/3/506/s1, Figure S1: SERS spectra of 5 mg/L fonofo in paddy water using GNRs obtained with addition of 40 (a), 60 (b) and 80 (c) μL 0.008 M AgNO_3_ during the phase of growth. Figure S2: SERS spectra of fonofos (A), phosmet (B) and sulfoxaflor (C) of different concentration in real paddy water. And the reference values were measured using GC-MS. Figure S3: UV–Vis spectrum of GNRs after deposition.Table S1: Pesticide residues in the contaminated paddy water samples. Table S2: Division of calibration set or validation set.

## Abbreviations

The following abbreviations are used in this manuscript:

SERS	surface-enhanced Raman spectroscopy
PLSR	partial least squares regression
SVM	support vector machine regression
KNN	K-near neighbour
RF	random forest
NB	Naive Bayes
CTAB	Cetyltrime-thylammonium bromide
GNR	gold nanorods
UV–Vis	ultraviolet-visible
SEM	scanning electron microscope
RMSE	root-mean-square error
R^2^	coefficient of determination
