# Supplementary material for: Rapid Detection of Pesticide Residues in Paddy Water Using Surface-Enhanced Raman Spectroscopy
Source: Sensors (Basel). 2019 Jan 26;19(3):506. doi: 10.3390/s19030506 (PMC6386844; doi:10.3390/s19030506)
Supplement: Supplementary file 1 [file sensors-19-00506-s001.pdf]

Support Information

Rapid Detection of Pesticide Residues in Paddy Water Using Surface-Enhanced Raman Spectroscopy

Shizhuang Weng [1,*], Wenxiu Zhu [1], Ronglu Dong [2], Ling Zheng [1,*] and Fang Wang [1]

[1] National Engineering Research Center for Agro-Ecological Big Data Analysis & Application, Anhui University, Hefei, 230601, China; zhuzwx1995@163.com (W.Z.); xyx_cm0018@163.com (F.W.)
[2] Institute of Intelligent Machines, Chinese Academy of Sciences, Hefei 230031, China; dongrl@mail.ustc.edu.cn
* Corresponding author: weng_1989@126.com (S.W.); lingz0865@163.com (L.Z.)

Abbreviations

The following abbreviations are used in this manuscript:

SERS	surface-enhanced Raman spectroscopy
PLSR	partial least squares regression
SVM	support vector machine regression
KNN	K-near neighbour
RF	random forest
NB	Naive Bayes
CTAB	Cetyltrime-thylammonium bromide
GNR	gold nanorods
UV–Vis	ultraviolet-visible
SEM	scanning electron microscope
RMSE	root-mean-square error
R^2	coefficient of determination

Synthesis of Au nanorods:

Seeds solution: 0.05 mL, 50 mM $HAuCl_4$ was injected into10 mL, 0.1 M CTAB. Then, 0.60 mL, 10 mM $NaBH_4$ was mixed with the solution.

Growth of Au nanorods: The $AgNO_3$ solution (0.008 M) of 40, 60 to 80 μL was added to the conical flask with 0.50 mL, 10 mM $HAuCl_4$ and 10 mL, 0.10 M CTAB, respectively. And PH of solution was adjusted with 0.1 mL, 2.0 M HNO_3. Then, 0.06 mL, 0.1 mL L-ascorbic acid was added for reduction of Au. At Last, 12 μL seed solution was added into the solution. After standing for 4 h, the Au nanorods of different aspect ratios (plasmon wavelengths) were obtained. All operations needed to be performed at 29 °C.

Gold nanorods (GNRs) of particular aspect ratios (plasmon wavelengths) were selected based on enhancement effect. With different addition of 0.008 M $AgNO_3$ during the phase of growth of Au nanorods, GNRs of different draw ratio can be obtained. From figure S1, GNRs obtained with the addition of 60 μL 0.008 M $AgNO_3$ showed the best enhancement, so the GNRs were selected the as active substrate for SERS in subsequent analysis.

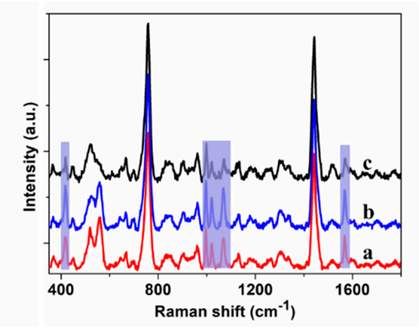

Figure S1. SERS spectra of 5 mg/L fonofo in paddy water using GNRs obtained with addition of 40 (a), 60 (b) and 80 (c) μL 0.008 M $AgNO_3$ during the phase of growth.

Measurement of real contaminated samples

The contaminated paddy water samples were obtained from Center of Agricultural Products Quality and Safety, Anhui Academy of Agricultural Sciences. Actual values were obtained using a GC-MS instrument (Thermo Fisher, TSQ8000EVO) and shown in Table S2. Spectra of real contaminated samples were measured and shown in Figure S2.

Table S1. Pesticide residues in the contaminated paddy water samples.

Pesticides	Reference Value Obtained by GC-MS (mg/L)
fonofos	9.73, 9.54, 4.76, 1.97, 1.05
phosmet	4.96, 2.21, 0.93, 0.55, 0.22
sulfoxaflor	10.03, 9.56, 4.86, 2.03, 0.95

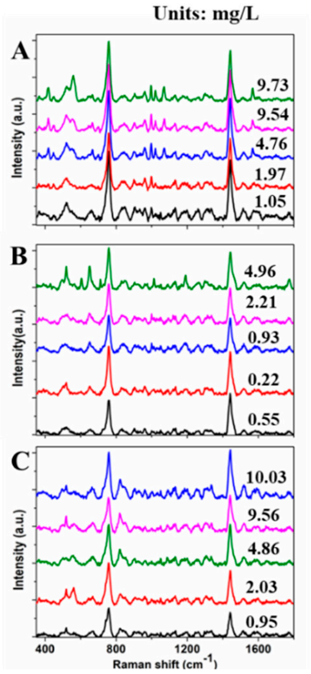

Figure S2. SERS spectra of fonofos (A), phosmet (B) and sulfoxaflor (C) of different concentration in real paddy water. And the reference values were measured using GC-MS.

Division of calibration set or validation set

Fifteen water samples were collected from Feixi rice-base in Hefei. Water samples were added with fonofos, phosmet and sulfoxaflor to obtain the contaminated samples, respectively. Fonofos concentration in paddy water is 10, 5, 2, 1 and 0.5 mg/L, and phosmet concentration is 10, 5, 2, 1, 0.5 and 0.25 mg/L, and sulfoxaflor concentration is 20, 15, 10, 5, 2 and 1 mg/L. Twelve water samples with pesticides of different concentration are selected as the calibration set, and the remaining water samples are used as validation set.

Table S2. Division of calibration set or validation set.

Calibration Set	Validation Set
Water samples #1, 2,…,12 with 10, 5, 2, 1 and 0.5 mg/L fonofos	Water samples #13, 14, 15 with 10, 5, 2, 1 and 0.5 mg/L fonofos
Water samples #1, 2,…, 12 with 10, 5, 2, 1, 0.5 and 0.25 mg/L phosmet	Water samples #13, 14, 15 with 10, 5, 2, 1, 0.5 and 0.25 mg/L phosmet
Water samples #1, 2,…, 12 with 20, 15, 10, 5, 2 and 1 mg/L sulfoxaflor	Water samples #13, 14, 15 with 20, 15, 10, 5, 2 and 1 mg/L sulfoxaflor

Absorption spectra of Au nanorods

After deposition, the plasmon wavelengths of Au nanorods were blueshifted to 506 and 622 nm from 517 and 636.

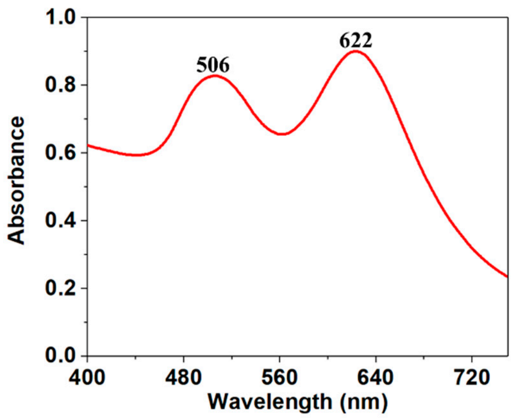

Figure S3. UV–Vis spectrum of GNRs after deposition.

Calibration of spectral band

In the synthesis of gold nanorods, CTAB is used to stabilize the high energy surface of the nanorods and protect them from aggregation. Gold nanorods is capped by CTAB though being washed by centrifuging. In SERS spectroscopy, the bands at 758 and 1439 cm^{-1} are assigned to the CTAB residue. Moreover, the bands of CTAB are so stable, and the peak at 758 cm^{-1} is chose as an internal standard for calibration of intensity of characteristic peaks. The detailed process is according to the following formula：

$$S' = \sum_{i=1}^{n} \frac{I_{758}}{\overline{I_{758}}} * S_i ,$$

Which, S_i is the intensity of the ith sampling point, and S' is the corrected spectrum. I_{758} is the intensity of peak at 758 cm^{-1} for the spectrum being corrected, and $\overline{I_{758}}$ is the mean intensity of peak at 758 cm^{-1} of all the obtained spectra. And the corrected spectra were used for the subsequent analysis.
